# ILC2 transfers to apolipoprotein E deficient mice reduce the lipid content of atherosclerotic lesions

**DOI:** 10.1186/s12865-019-0330-z

**Published:** 2019-12-10

**Authors:** Polyxeni T. Mantani, Pontus Dunér, Irena Ljungcrantz, Jan Nilsson, Harry Björkbacka, Gunilla Nordin Fredrikson

**Affiliations:** 1 Department of Clinical Sciences, Skåne University Hospital Malmö, CRC, Building 91:12, Jan Waldenströms gata 35, 20502 Malmö, Sweden; 2 Lund University, Lund, Sweden

**Keywords:** ILC2s, IL-25, Apolipoprotein E deficient mice, B1 cells, Anti-PC IgM, Eosinophils, Alternatively activated macrophages, Atherosclerosis

## Abstract

**Background:**

Expansion of type 2 innate lymphoid cells (ILC2s) in hypercholesterolaemic mice protects against atherosclerosis while different ILC2 subsets have been described (natural, inflammatory) based on their suppression of tumorigenicity 2 (ST2) and killer-cell lectin like receptor G1 (KLRG1) expression. The aim of the current study is to characterize the interleukin 25 (IL25)-induced splenic ILC2 population (Lin^−^CD45^+^IL17RB^+^ICOS^+^IL7ra^intermediate^) and address its direct role in experimental atherosclerosis by its adoptive transfer to hypercholesterolaemic apolipoprotein E deficient (apoE^−/−^) mice.

**Results:**

Immunomagnetically enriched, FACS-sorted ILC2s from the spleens of IL-25 treated apoE^−/−^ mice were stained for KLRG1 and ST2 directly upon cell obtainment or in vitro cell expansion for flow cytometric analysis. IL25-induced splenic ILC2s express high levels of both KLRG1 and ST2. However, both markers are downregulated upon in vitro cell expansion. In vitro expanded splenic ILC2s were intraperitoneally transferred to apoE^−/−^ recipients on high fat diet. ApoE^−/−^ mice that received in vitro expanded splenic ILC2s had decreased lipid content in subvalvular heart and brachiocephalic artery (BCA) plaques accompanied by increased peritoneal B1 cells, activated eosinophils and alternatively activated macrophages (AAMs) as well as anti-phosphorylcholine (PC) immunoglobulin (Ig) M in plasma.

**Conclusions:**

With the current data we designate the IL25-induced ILC2 population to decrease the lipid content of atherosclerotic lesions in apoE^−/−^ mice and we directly link the induction of B1 cells and the atheroprotective anti-PC IgM antibodies with ILC2s.

## Background

Atherosclerosis is a lipid-driven, inflammatory disease characterized by the accumulation of lipids in the arterial wall. Local lipoprotein retention leads to the modification of low-density-lipoprotein (LDL) into oxidized-LDL (oxLDL), a term denoting several versions of the LDL particle bearing modifications of the lipids and/or its structural protein apolipoprotein B (apoB). These modifications are targeted by the immune system as non-self, leading to the induction of immune responses against the newly generated oxidation-related neo-epitopes. In a crude categorization, T effector immune responses in experimental studies have been shown to be either disease-promoting (T helper 1, Th1) [1–4] or athero-protective when merely focusing on the Th2-related cytokines IL-5 [5] and IL-13 [6], while the role of Th17 immune responses is still under debate [7]. T regulatory (Treg) immune responses are considered to protect from disease progression and increasing Tregs is considered as a possible therapeutic pathway [8]. Focusing on the humoral part of innate immunity, B1 cell-derived natural immunoglobulin (Ig) M antibodies that target oxLDL have been shown to be protective both in mice [9, 10] and humans [11, 12]. Splenectomized mice have drastically decreased levels of peritoneal B1 cells [13] indicating that the spleen is an important provider of survival signals for B1 cells while splenectomy in apoE^−/−^ mice leads to increased atherosclerosis [14].

The onset for the discovery of type 2 innate lymphoid cells (ILC2s) came by studies showing that administration of interleukin-25 (IL-25) in Rag^−/−^ mice that lack functional B and T cells led to the induction of IL-5 and IL-13 [15, 16]. In 2010 the existence of ILC2s was established by three independent groups [17–19] where ILC2s were reported to expand upon helminthic infection or administration of exogenous IL-25 and IL-33 and to secrete large amounts of IL-5 and IL-13. Interestingly, in one of the studies ILC2s were reported to support B1 cell survival and antibody secretion while it was suggested that this could be done through the secretion of IL-5 and IL-6, supporting the idea of the existence of a unique pathway of interplay between innate and adaptive immunity [17]. The interaction of ILC2s with T cells was also reported by a later study showing that MHC class II expressing ILC2s proliferate in response to IL-2 derived from antigen-specific T cells [20]. ILC2s have been reported to be pathologically involved in allergy and asthma but to promote wound healing and metabolic homeostasis in visceral adipose tissue (VAT) (reviewed in [21]). The maintenance of metabolic homeostasis in VAT has been assigned to the existence of ILC2s at that site leading to the recruitment of eosinophils through ILC2-cytokine secretion and the induction of alternatively activated macrophages (AAMs) [22].

Differential subsets of ILC2s have also been reported to be induced by IL-25 and IL-33. IL-25 was reported to induce among others a specific type of ILC2s called inflammatory ILC2s (iILC2s) in lung tissues characterized by high levels of killer-cell lectin like receptor G1 (KLRG1) and low levels of suppression of tumorigenicity 2 (ST2) that can “sense” the micro-environment and secrete cytokines (type 2 or IL-17) accordingly [23]. IL-33 was shown to mainly expand the natural ILC2s (nILC2s) that express high levels of ST2 and low levels of KLRG1 representing the tissue resident ILC2s [23]. Interestingly, the IL25-induced iILC2s were shown to serve as progenitors of nILC2s [23].

Only a few studies have touched upon the possible role of ILC2s in atherosclerosis until now. Perry et al. have reported the existence of ILC2s and B1a cells in aortic mouse specimens and identified Id3 as a regulator of B1a cells and IL-5 production from ILC2s [24]. Our group published the very first study to implicate ILC2s in experimental atherosclerosis and atheroprotection by showing that treatment of apoE^−/−^ mice with IL-25 reduced atherosclerosis through the induction of splenic ILC2s, IL-5, B1 cells and anti-phosphorylcholine (PC) IgM antibodies that target oxLDL [25]. Engelbertsen et al. have shown in a hypercholesterolaemic mouse model lacking adaptive immunity that treatment with IL-2/anti-IL2 complexes expands CD25^+^ ILCs leading to decreased plaque size, a phenomenon that was accompanied by reduced cholesterol levels in serum and eosinophilia [26]. With the use of bone marrow chimeras Newland et al. have shown that genetic deletion of ILC2s in LDLr^−/−^ mice exacerbates atherosclerosis and that this could be prevented by reconstitution with wild type but not IL5^−/−^ or IL13^−/−^ ILC2s [27].

In the present study we attempt to further characterize the splenic IL25-induced ILC2 subset (Lin^−^CD45^+^IL17RB^+^ICOS^+^IL7ra^intermediate^) as well as investigate the cells subset’s direct effect on atherosclerosis development through its adoptive transfer to atherosclerosis-prone apoE^−/−^ mice. For that reason we have immunomagnetically enriched and FACS-sorted splenic ILC2s (as previously shown [25]) from IL-25 treated apoE^−/−^ mice, expanded them in vitro and transferred them i.p. to apoE^−/−^ recipient mice placed on high fat diet. We observed that mice that received ILC2s had decreased lipid content in subvalvular heart and BCA lesions accompanied by increased levels of peritoneal B1 cells, activated eosinophils and AAMs as well as anti-PC IgM in plasma.

## Results

### ILC2 characterization

**Fig. 1:**
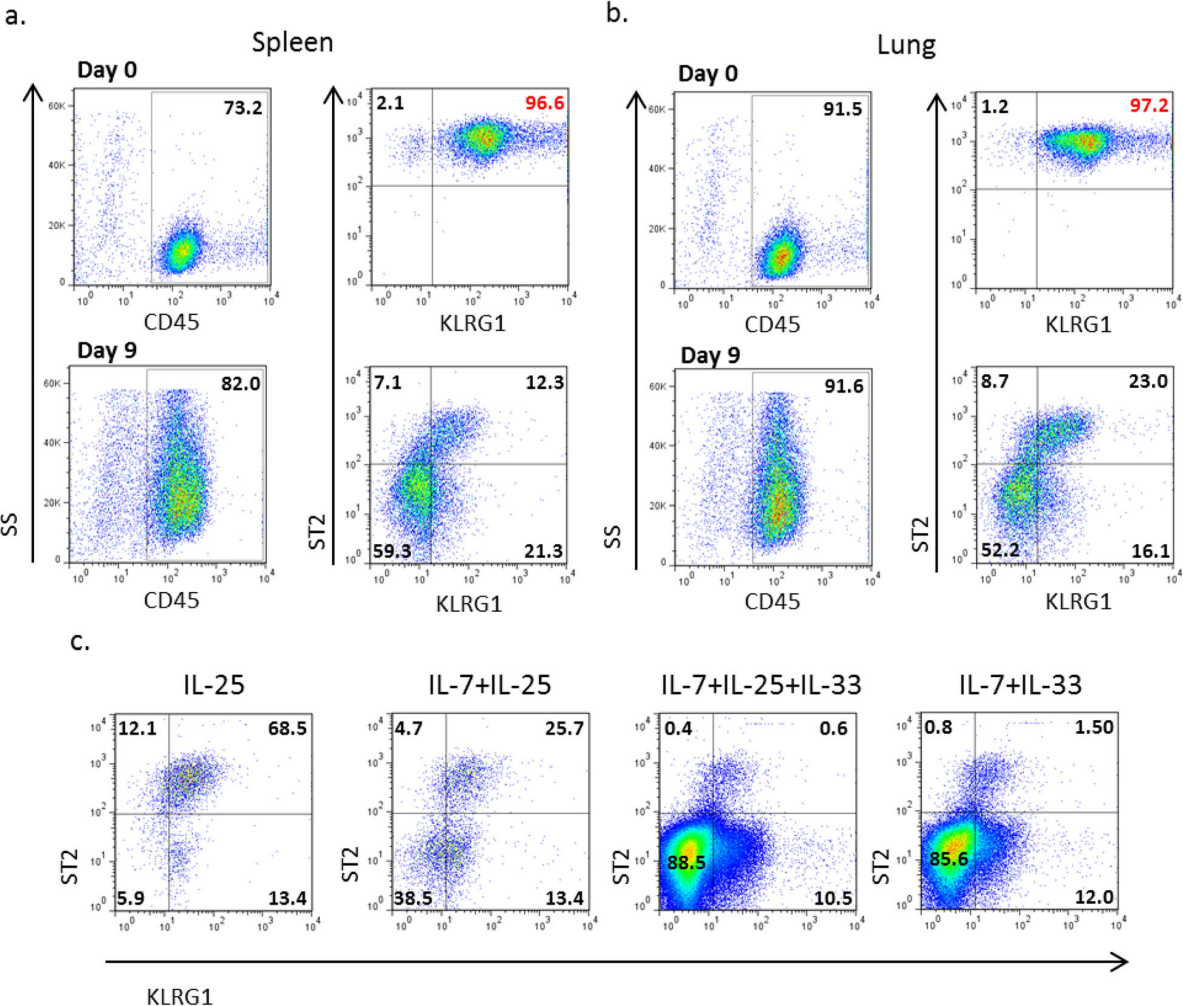
KLRG1 and ST2 expression of IL25-induced ILC2s. FACS-sorted ILC2s (Lin^−^CD45^+^IL17RB^+^ICOS^+^IL7ra^int^) from pooled splenocytes and lung cells of IL25-injected *apoE*^*−/−*^ mice (*n* = 8–9) were cultured in the presence of IL-7 (10 ng/ml) and IL-33 (10 ng/ml). Cells were next stained with fluororochrome conjugated antibodies targeting CD45, KLRG1 and ST2 for flow cytometric analysis. Representative FACS plots are depicted for in vitro expanded ILC2s of splenic (**a**) and lung origin (**b**), number of experiments, *n* = 2. Part of splenic FACS-sorted ILC2s were also incubated with different combinations of ILC2-expanding cytokines (10 ng/ml each) (**c**) and stained with fluorochrome conjugated antibodies targeting CD45, KLRG1, ST2. Representative FACS plots of in vitro expanded ILC2s in the presence of IL-25, IL7 + IL25, IL7 + IL25 + IL33, IL7 + IL33 are indicated in (**c**). In all cases, numbers on plots represent percentages of CD45^+^ gated cells

In order to investigate whether splenic IL25-induced ILC2s (Lin^−^CD45^+^IL17RB^+^ICOS^+^IL7ra^int^) could be categorized as inflammatory or natural ILC2s we assessed their expression of KLRG1 and ST2 markers as has previously been shown with lung ILC2s [23]. For that reason we injected apoE^−/−^ mice (*n* = 8–9) with IL-25 (1 μg/day) for a week and sorted ILC2s from pooled spleen and lung cells, number of experiments, *n* = 2. ILC2s were immunomagnetically enriched and FACS-sorted as Lin^−^CD45^+^IL17RB^+^ICOS^+^IL7ra^int^ according to Additional file 1 a. Part of the sorted cells were directly stained with fluorochrome-conjugated antibodies targeting CD45, KLRG1 and ST2 while the rest were incubated in vitro (0.8 × 10^4^ ILC2s/ml) with cytokines (IL-7, IL-33) that were previously shown to optimally expand ILC2s [18]. It appears that the FACS-sorted ILC2s which we previously showed to be implicated in athero-protection [25] express high levels of both KLRG1 and ST2 (red numbers in Fig. 1a) thus not falling into the exact same category as iILC2s or nILC2s. Moreover, when we compared cells sorted either from the spleen or lungs we did not observe different patterns of KLRG1 and ST2 expression showing that the tissue origin of the cells does not play a role (Fig. 1a, b). However, when the cells were incubated in cell culture medium supplemented with IL-7 and IL-33 the expression levels of KLRG1 and ST2 changed drastically with the main body of the cells being negative for those markers (lower FACS plots, Fig. 1a, b). Thus, IL-7, IL-33- expanded ILC2s differ in terms of KLRG1 and ST2 expression than the ones that are obtained upon sorting after a week of daily IL-25 administration to apoE^−/−^ mice (1 μg/day).

We next incubated splenic FACS-sorted ILC2s (Lin^−^CD45^+^IL17RB^+^ICOS^+^IL7ra^int^, 10^4^ cells/ml/well) with different combinations of ILC2-inducing cytokines in order to see how the expression levels of KLRG1 and ST2 fluctuate (Fig. 1c). All samples were run as a free run in the flow cytometer upon harvesting of the cells from each well that were initially seeded in equal numbers (Fig. 1c). The presence of IL-7 and IL-33 and in particular their combination, although optimally expands ILC2s it also drastically changes the expression levels of KLRG1 and ST2 (Fig. 1c). The presence of IL-25 only preserves the expression levels of KLRG1 and ST2 but it does not yield a big number of ILC2s (Fig. 1c). We previously reported that the IL-7, IL-33-expanded cell population increases the levels of B1a cells in the spleen of apoE^−/−^ mice [25]. Thus, although the expansion of ILC2s in the presence of IL-7 and IL-33 drastically changes the expression levels of KLRG1 and ST2 differentiating them from the IL25-induced ILC2 population obtained upon sorting, the expanding conditions lead to a satisfying number of ILC2s that are functional in vivo when transferred to apoE^−/−^ mice. Additionally, we previously reported that the main part of the transferred ILC2s stay in the peritoneal cavity while part of them migrate to the spleen [25]. Due to the fact that the peritoneal cavity is the main residence of B1 cells we decided to transfer IL-7, IL-33-expanded ILC2s intraperitoneally to apoE^−/−^ mice and study the induced immunological response as well as their effect on atherosclerosis.

### Peripheral induced immune response upon ILC2 transfers

**Fig. 2:**
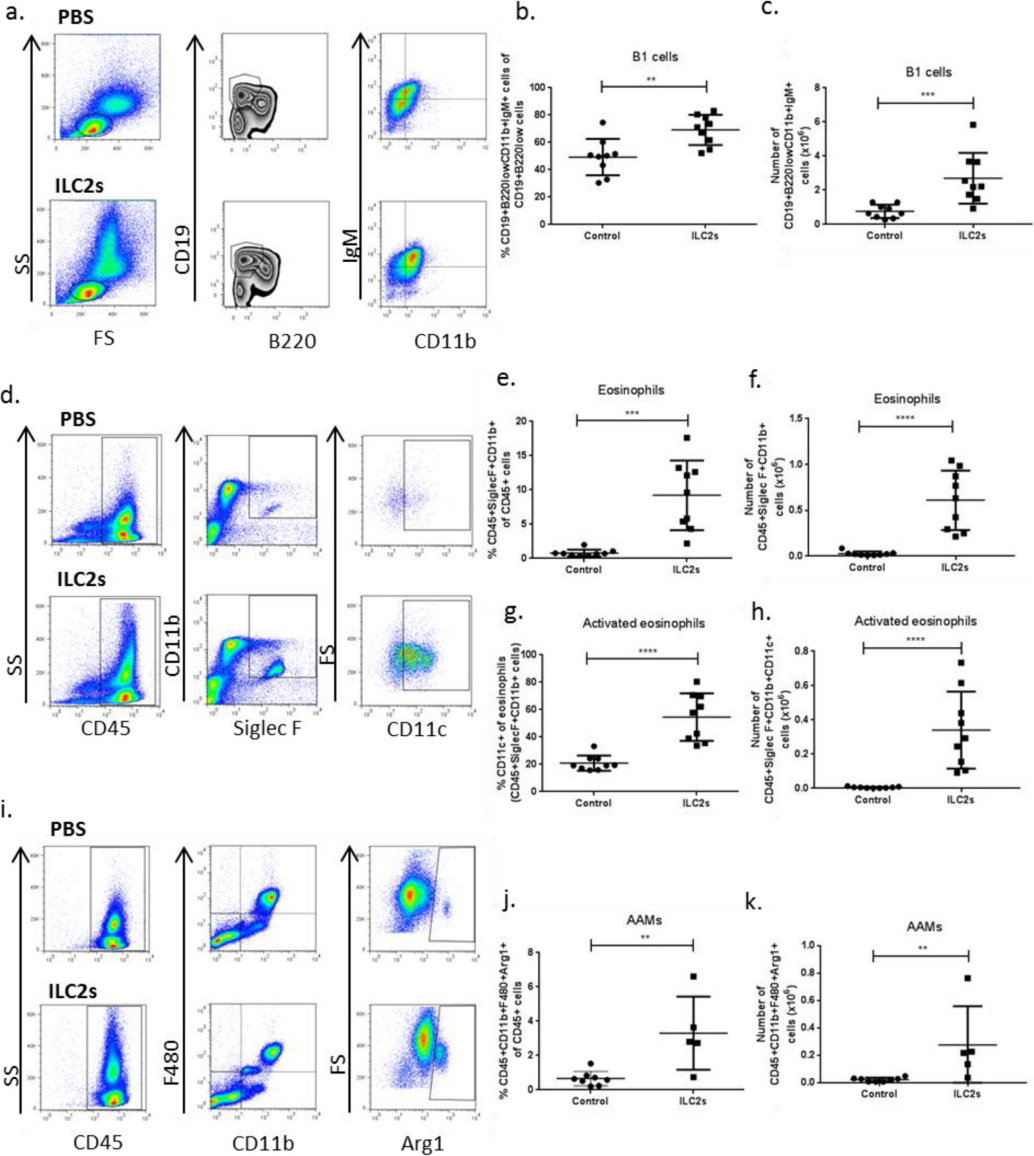
ILC2 transfers to apoE^−/−^ mice increase peritoneal B1 cells, eosinophils and AAMs. Flow cytometry gating strategies of B1 cells identified as CD19^+^B220^low^CD11b^+^IgM^+^ (**a**), eosinophils as CD45^+^SiglecF^+^CD11b^+^ cells (**d**) and AAMs as CD45^+^CD11b^+^F480^+^Arg1^+^ cells (**i**) of apoE^−/−^ mice for both control and ILC2 groups respectively as indicated. Percentages and numbers of peritoneal B1 cells (**b**, **c**), eosinophils (**e**, **f**) and AAMs (**j**, **k**). CD11c^+^ expression from gated eosinophils and respective cell numbers of activated eosinophils identified as CD45^+^SiglecF^+^CD11b^+^CD11c^+^cells (**g**, **h**). Mann Whitney *U* test, **P < 0.01, ***P < 0.001, ****P < 0.0001. Each data point represents one mouse

**Fig. 3:**
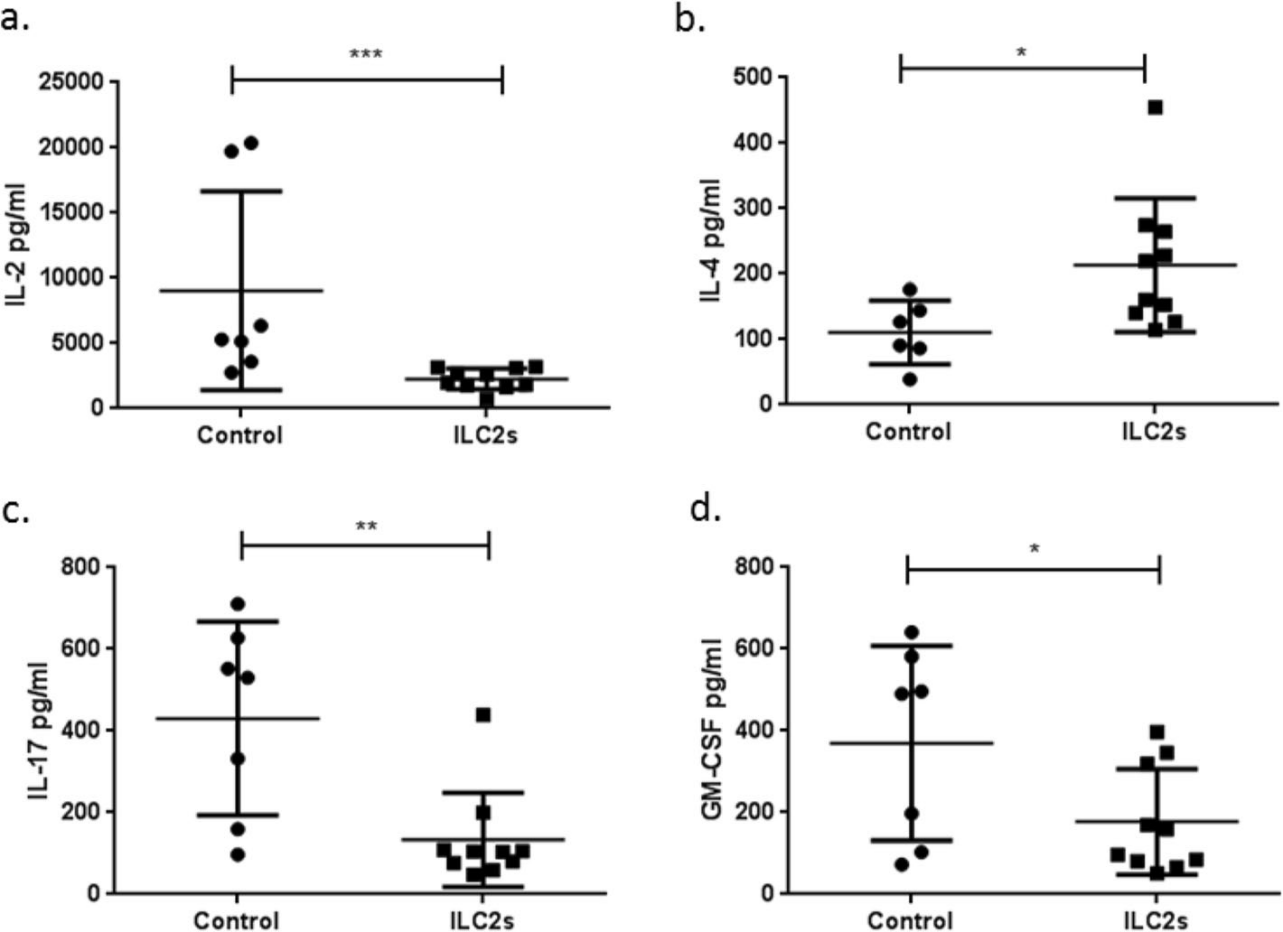
ILC2 transfers to apoE^−/−^ mice induce an anti-inflammatory cytokine profile from peritoneal cells. Cytokine expression levels of IL-2 (**a**), IL-4 (**b**), IL-17 (**c**), GM-CSF (**d**) from peritoneal cells of apoE^−/−^ mice stimulated in vitro with PMA and Ionomycin. Mann Whitney *U* test *P < 0.05, **P < 0.01, ***P < 0.001. Each data point represents one mouse

ApoE^−/−^ mice (7–8 weeks old) received in total 4 intraperitoneal (i.p.) transfers of FACS-sorted (Additional file 1 a) and in vitro expanded ILC2s (0.5 × 10^6^) in PBS or equal volume of PBS only as control while on high fat diet according to Additional file 1 b. Initially we were interested in studying the immunological response that the transfer of ILC2s evoked in the peritoneal cavity, spleen and blood of apoE^−/−^ mice. Transfer of ILC2s in the peritoneal cavity increased the levels of B1 cells (Fig. 2a-c), eosinophils (Fig. 2d-h) and AAMs (Fig. 2i-k). Increased numbers of CD45^+^Arginase (Arg) 1^+^ cells (Mean ± St Dev, 0.55 ± 0.19 × 10^6^, *n* = 8, control group vs 2.30 ± 0.80 × 10^6^, *n* = 5, ILC2 group, *P* = 0.002, Mann Whitney *U* test) were observed in the group of mice that received ILC2s indicating that a big part of the cells resided in the peritoneal cavity since Arg1 has been shown to be expressed by mature ILC2s [28] and since this increase could not be merely accounted for by the increase of AAMs (Fig. 2i-k). We also evaluated the cytokine secretion levels of peritoneal cells when we stimulated them in vitro in the presence of PMA and Ionomycin. IL-2 (Fig. 3a), IL-17 (Fig. 3c) and granulocyte macrophage colony-stimulating factor (GM-CSF) (Fig. 3d) cytokine production were reduced, IL-4 (Fig. 3b) was increased while no other differences were found for the rest of the cytokines tested (Additional file 2). The increased IL-4 levels possibly reflect the robust accumulation of eosinophils as previously shown [29].

**Fig. 4:**
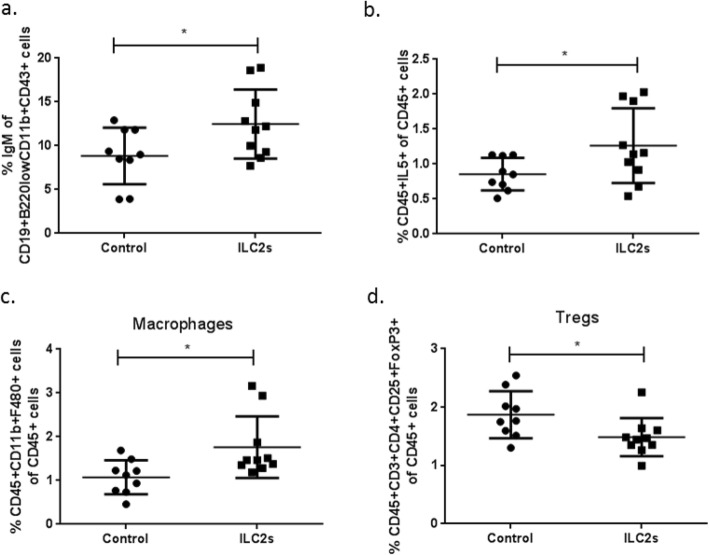
ILC2 transfers to apoE^−/−^ mice affect B1 cells, macrophages and Tregs in the spleen. IgM expression levels from splenic B1 cells of apoE^−/−^ mice gated as CD19^+^B220^low^CD11b^+^CD43^+^ cells (**a**). Percentages of splenic CD45^+^IL-5^+^ cells (**b**) macrophages gated as CD45^+^CD11b^+^F480^+^ cells (**c**) and Tregs identified as CD45^+^CD3^+^CD4^+^CD25^+^FoxP3^+^ cells (**d**) of the CD45^+^ cell population. All cell populations were assessed by flow cytometry. Mann Whitney *U* test, *P < 0.05. Each data point represents one mouse

**Fig. 5:**
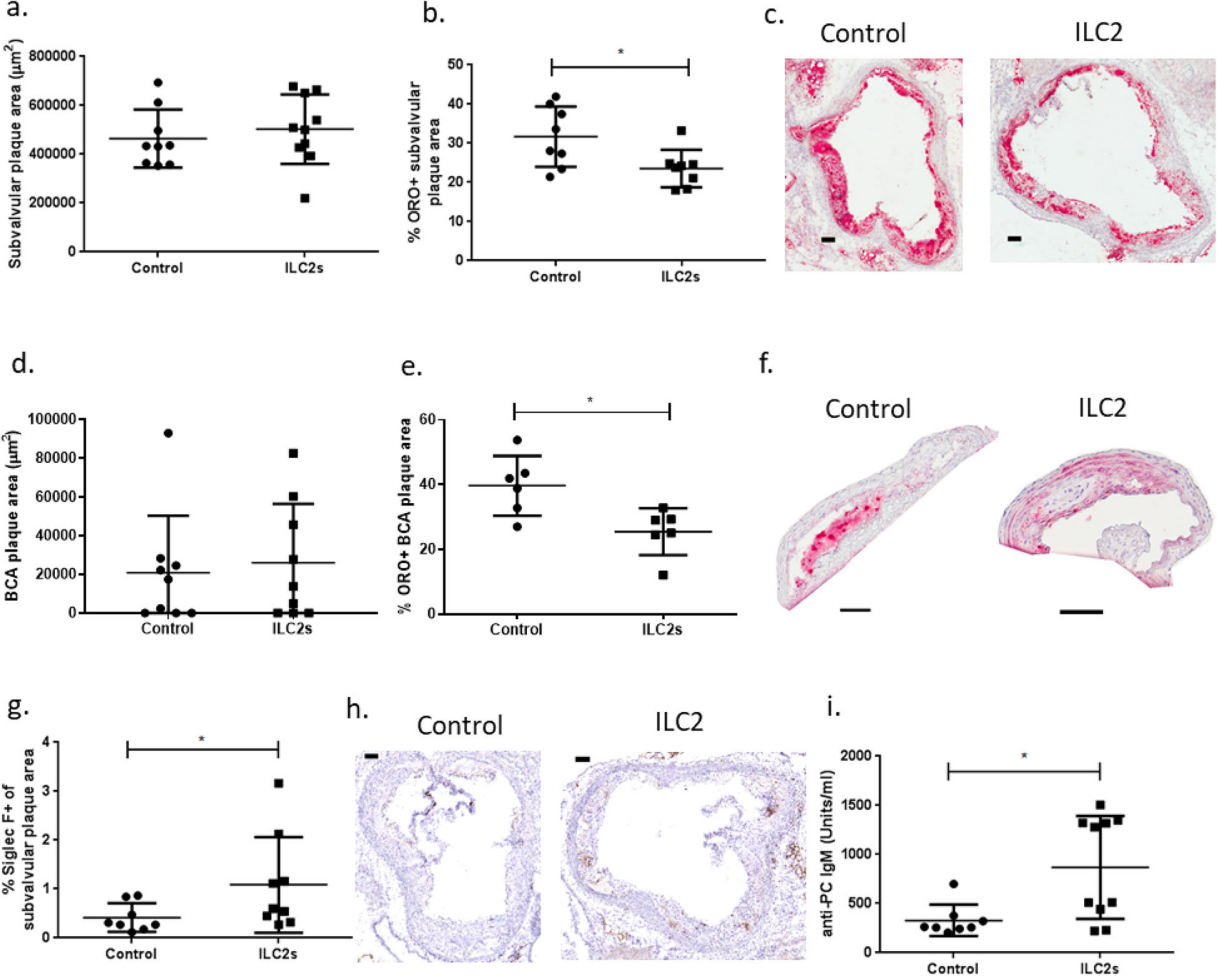
ILC2 transfers to apoE^−/−^ mice reduce the lipid content of atherosclerotic lesions. Quantification of atherosclerotic lesions of hematoxylin/eosin stained subvalvular heart (**a**) and brachiocephalic artery (BCA) sections (**d**) of apoE^−/−^ mice fed a high fat diet for 9 weeks. The mice received 4 i.p. ILC2 transfers (0.5 × 10^6^ cells/transfer) or equal volume of PBS during that time period until euthanasia at 16–17 weeks of age. Neutral lipid content of subvalvular heart (**b**, **c**) and BCA (**e**, **f**) sections of apoE^−/−^ mice that received ILC2s (or PBS as a control) performed with Oil Red O (ORO) staining and respective representative pictures of stainings (**c**, **f**). Siglec F (**g**) content in subvalvular heart sections of apoE^−/−^ mice that received ILC2s (or PBS as a control) and representative immunohistochemistry pictures (**h**) for both groups. Scale bar 100 μm in all cases. Anti-PC IgM levels in the plasma of mice that received ILC2s or PBS as a control (**i**). Mann Whitney *U* test, *P < 0.05. Each data point represents one mouse

ApoE^−/−^ mice that received ILC2s had increased IgM expression levels from B1 cells (Fig. 4a) as well as increased levels of IL5^+^ expressing CD45^+^ cells (Fig. 4b) in the spleen possibly indicating migration of ILC2s from the peritoneal cavity at that location as we have previously shown [25]. Moreover, for splenocytes that have been extracted and stimulated in vitro with PMA and Ionomycin increased levels of IL-5 were observed for the group of mice that received ILC2s (Mean ± St Dev, 169.3 ± 97.6, *n* = 9 for control vs 312.7 ± 258.8 pg/ml, *n* = 10 for the ILC2 group, *P* = 0.04, Mann Whitney *U* test). No other statistically significant differences were observed for the rest of the cytokines tested (Additional file 3). Additionally, increased levels of macrophages (gated as CD45^+^CD11b^+^F480^+^, Fig. 4c) and decreased levels of Tregs (Fig. 4d) in the spleen were also recorded for the group of mice that received ILC2s. Overall it seems that the most pronounced immunological differences were observed in the peritoneal cavity which probably indicates that the main body of ILC2s resided at that location. Plasma cytokine (IL-1β, IL-2, IL-4, IL-5, IL-6, IL-9, IL-10, IL-12(p70), IL-13, IL-17A, Eotaxin, GM-CSF, interferon γ (IFNγ); Additional file 4) and immunoglobulin (IgA, IgG1, IgG2a, IgG2b, IgM; Additional file 5) levels were assessed without detection of any statistical significant differences. However, the group of apoE^−/−^ mice that received ILC2s had increased plasma levels of IgM antibodies targeting PC which is an oxLDL epitope (Fig. 5i).

### ILC2 transfers - effect on atherosclerosis

In order to assess the effect of ILC2 transfers on atherosclerosis outcome, hearts and brachiocephalic arteries were dissected, sectioned and stained for their neutral lipid content (Oil Red O staining). Transfer of ILC2s to apoE^−/−^ mice resulted in decreased lipid content of atherosclerotic plaques in both subvalvular heart (Fig. 5b, c, *P* = 0.02, *t*-test) and BCA sections (Fig. 5e, f, *P* = 0.02, Mann Whitney *U* test) without affecting the size of the plaques (Fig. 5a, d) nor necrotic core areas (Additional file 6). In sections of the BCA arteries no necrotic cores could be observed. No difference in aortic plaque area was observed (% Oil Red O^+^ of whole aortic area, Mean ± St Dev, 1.2 ± 0.5, *n* = 9 for control vs 1.5 ± 0.7, *n* = 10 for the ILC2 group, *P* = 0.21, Mann Whitney *U* test). No difference in total cholesterol (Additional file 7a), LDL/VLDL cholesterol (Additional file 7 b), HDL cholesterol (Additional file 7 c) and triglyceride (Additional file 7 d) levels was observed that could account for the decreased lipid content in plaques and also there was no difference in the weight of mice (Additional file 7 e). We also focused on plaque composition analysis targeting the macrophage (CD68), CD3, α actin smooth muscle cell (αSMactin), arginase 1, Siglec F and IgM content. In subvalvular heart sections we detected increased eosinophil content (Fig. 5g, h) but no statistically significant differences occurred for the rest of the markers that were tested (Additional file 8 a-f). In BCA sections, no differences were detected in macrophage (CD68), CD3, αSMactin, IgM (Additional file 9 a-d) content while we could not detect any stained areas positive for arginase I and Siglec F. In conclusion, transfer of intraperitoneal transfer of ILC2s in apoE^−/−^ mice affected atherosclerosis by reducing the lipid content of the lesions formed.

## Discussion

In the present study we attempted to investigate the direct effect of the IL25-induced splenic ILC2 (Lin^−^CD45^+^IL17RB^+^ICOS^+^IL7ra^int^) population on the development of atherosclerosis by its adoptive transfer to hypercholesterolaemic apoE^−/−^ mice. We found that apoE^−/−^ mice that received ILC2s had decreased lipid content in both subvalvular heart and BCA lesions as well as increased peritoneal B1 cells and plasma anti-PC IgM levels. Since no altered lipid profile was observed for apoE^−/−^ mice that received ILC2s it is possible that the induction of IgM antibodies targeting oxLDL contributed to the decreased lipid content of atherosclerotic lesions. The current data are in accordance with the IL-25 study where we showed that the expansion of ILC2s and the net effect of B1 cell induction and anti-oxLDL IgM increase are responsible for the administered cytokine’s athero-protective effect [25]. However, in our IL-25 study we recorded decreased atherosclerosis burden in whole aortas while in the present study we could only detect decreased lipid content in the plaques of mice that received ILC2s. Although this is a limitation of the current study, this discrepancy can be a matter of magnitude; exogenous IL-25 administration most likely induces expansion of ILC2s in several tissues leading to their robust activation, secretion of IL-5 levels and enhanced induction of anti-PC IgM. The biological effect of such activation most likely cannot be compared to the transfer of a limited number of ILC2s in the peritoneal cavity. This approach efficiently led to the induction of B1 cells and anti-PC IgM antibody production and decreased the lipid content of the lesions formed. However, we cannot exclude the possibility that multiple mechanisms could have taken place upon IL-25 administration in apoE^−/−^ mice [25] that could account for the discrepancy in terms of disease burden between the two studies. Moreover, the phenotypic changes of the transferred ILC2s could be accountable for not detecting any differences in plaque size which is a limitation of the current study. Although we believe that the in vitro phenotypic changes of the transferred ILC2s could be to a large extent due to their in vitro culture during the expansion period and in extension due to lack of additional signals existing at the relative in vivo environment, we cannot however exclude this possibility. Thus, we consider all the above as limitations of the current study.

Plaques prone to rupture, characterized as vulnerable, are highly inflammatory with increased lipid and cellular content. Atherosclerotic plaques with high lipid content are associated with increased incidence of rupture and thrombosis [30, 31] indicating that it is of clinical importance the formation of more stable plaques by targeting such destabilizing plaque features. In this context, the current study is providing evidence that intraperitoneal ILC2 transfers to apoE^−/−^ mice affect lesion composition by decreasing the lipid content of the plaques formed. However, we were not able to find any other differences in the major cellular (macrophage, smooth muscle cell) content of the plaques formed. In experimental studies IgM antibodies targeting oxLDL are reported to have a protective role in atherosclerosis [9, 10] while in clinical studies there is an inverse association of anti-oxLDL IgM and cardiovascular disease [11, 12]. A limitation of the present study is that we do not directly prove that the decreased lipid content of the lesions formed upon ILC2 transfers to apoE^−/−^ mice is due to the induction of peripheral anti-PC IgM antibodies however we believe that this is an interesting possibility.

Intraperitoneal transfer of ILC2s besides the induction of B1 cells increased the number of eosinophils and AAMs at that location. The induction of eosinophils and AAMs is in line with an experimental study describing the induction of this particular mechanism in VAT upon IL-33 administration [22]. Our study shows that this mechanism can be induced directly by IL25-induced ILC2s and in a different location than adipose tissue. Moreover we detected increased eosinophil accumulation in subvalvular heart lesions of apoE^−/−^ mice that received ILC2s. A possible link between ILC2s and eosinophils has also been reported by another study showing that treatment of hypercholesterolemic mice lacking adaptive immunity with IL-2/anti-IL2 complexes led to the induction of CD25^+^ILCs, IL-5 and to a vast increase of eosinophils [26].

In atherosclerotic plaques, macrophages represent the major immune cell type. Regardless of their origin (recruited circulating monocytes, resident precursors or local proliferation) macrophages are plastic cells that are “shaped” by the local micro-environment. Classically activated macrophages (M1) secrete pro-inflammatory factors in response to toll-like receptor and interferon signaling and are enriched in advanced plaques while M2 macrophages (or AAMs) that are induced in response to IL-4 and IL-13, are involved in tissue repair and are located in more stable regions in the plaque (reviewed in [32]). In our study we could detect AAMs to increase in the peritoneal cavity upon transfer of ILC2s. However, we did not record increased arginase 1 expression in subvalvular heart or brachiocephalic lesions that could possibly indicate a local shift of the resident macrophages into AAMs or a possible migration of peritoneal AAMs to the lesions.

Cytokine secretion analysis from peritoneal cells showed decreased levels of IL-2, GM-CSF and IL-17 and an increase in IL-4 levels. Interestingly, GM-CSF has been reported to polarize macrophages into M1 which secrete pro-inflammatory cytokines, leading to the induction of Th17 immune responses [33, 34]. The decreased released levels of GM-CSF and IL-17 from peritoneal cells could possibly reflect the inhibition of that immune pathway. It has previously been reported that eosinophils is the main IL-4 expressing cell population in VAT while when these cells are depleted, AAMs are decreased [29]. The increased levels of IL-4 most likely reflect the vast induction of eosinophils that we observe in the peritoneal cavity and in extension the induction of AAMs. Moreover, IL-2 cytokine production is decreased from peritoneal cells that have been in contact with ILC2s. ILC2s expand in response to IL-2 and it has previously been suggested that IL-2 consumption by Tregs can control ILC2 homeostasis in tissues [21]. In apoE^−/−^IL25^−/−^ mice we consistently record decreased numbers of ILC2s in the spleen and in parallel an increased secretory capacity of IL-2 from in vitro stimulated splenocytes [35]. In the present study, the presence of ILC2s in the peritoneal cavity leads to decreased IL-2 secretory levels from peritoneal cells possibly showing an inhibitory effect (direct or indirect) of the ILC2s on other cell populations, possibly on T cells as previously suggested [20]. However, one limitation of the present study is that we did not investigate the T cell populations in the peritoneal cavity in order to prove such a link. However, Tregs were decreased in the spleen of apoE^−/−^ mice that received ILC2s. We have previously shown that upon intraperitoneal transfer of ILC2s in apoE^−/−^ mice, a fraction of cells migrate to the spleen [25]. The migrated ILC2 cell population could possibly account for the suppression of the Treg cell population through local IL-2 consumption. Moreover, in the spleen we detected increased release of IL-5 from in vitro stimulated splenocytes as well as increased IgM expression from B1 cells that might have contributed to the increased circulating anti-PC IgM levels.

In the current study we report reduced lipid content in subvalvular heart and BCA sections, increased plasma anti-PC IgM and reduced levels of Th17-associated cytokine secretion from peritoneal cells in apoE^−/−^ mice that received ILC2s. We have previously indicated that genetic deletion of IL-25 in apoE^−/−^ mice aggravates atherosclerosis in the aortic arch which was accompanied by increased levels of Th1 and Th17 responses as well as reduced frequencies of splenic ILC2s and plasma anti-PC IgM levels [35]. Based on the above, it appears that IL-25 and in extension its main target cell population, ILC2s, are implicated in a delicate balance between type 2 and Th1/Th17 immune responses by shifting the atherosclerotic burden depending on the cytokine’s absence or exogenous administration.

## Conclusions

In the present study we designate the investigated IL25-induced splenic ILC2 population as a cell population that upon its adoptive transfer to hypercholesterolaemic mice decreases the lipid content of the atherosclerotic lesions formed. Moreover, we directly link the induction of B1 cells and the atheroprotective anti-PC IgM antibodies with ILC2s. Although the translation of mouse data to humans is not always direct we believe that the induction of analogous mechanisms in humans has therapeutic potential targeting atherosclerosis.

## Methods

### Mice – ILC2s

For each cell transfer, 15 weeks old female apolipoprotein E deficient mice (*n* = 10) on C57BL/6 background (B6129P2-Apoe*tm1Unc*/J, Jackson Laboratories, Scanbur, Denmark) were daily injected with 1 μg of recombinant mouse interleukin-25 (rmIL-25, R&D Systems) for 7 days. Upon termination of the mice by carbon dioxide inhalation according to the NIH guidelines, spleens were dissected and ILC2s were immuno-magnetically enriched, FACS-sorted (as Lin^−^CD45^+^IL17RB^+^ICOS^+^IL7ra^int^, Additional file 1 a) and in vitro expanded in the presence of IL-7 and IL-33 as we previously described [25].

Female apoE^−/−^ mice (B6129P2-Apoe*tm1Unc*/J, Jackson laboratories, Scanbur, Denmark), 7–8 weeks old, were placed on high fat diet (HFD; 0.15% cholesterol and 21% fat (Lantmännen, Sweden)) for 9 weeks. Two weeks upon placement on HFD, serial i.p. transfers of in vitro expanded splenic ILC2s (0.5 × 10^6^ cells resuspended in PBS or PBS only as a control) took place 2 weeks apart from each other for a total of 4 transfers as shown in Additional file 1 b (number of mice; *n* = 9 for PBS group, *n* = 10 for ILC2 group). One week after the last transfer of ILC2s the mice were euthanized by i.p. injection of ketamine (50 μg/g mouse weight) and xylazine (10 μg/g mouse weight). Upon termination the mice were weighed, blood samples were taken by cardiac puncture and the obtained plasma was stored at − 80 °C. Peritoneal lavage was performed, spleens, hearts and brachiocephalic arteries and aortas were dissected. Mice were whole body perfused with PBS and BCA segments (above the bifurcation down to the branch of the aorta) were dissected and frozen at − 80 °C.

In control experiments (*n* = 2) targeting spleen and lung ILC2 characterization, 10–20 weeks old female apoE^−/−^ mice (*n* = 8–9) were injected daily with 1 μg of rmIL-25 (R&D Systems, Abingdon, UK) for 7 days and terminated by carbon dioxide inhalation according to the NIH guidelines. Upon termination, spleens and lungs were dissected and single cell suspensions from all mice were pooled for each tissue and processed for ILC2 isolation as previously described [25]. Concerning lung ILC2 isolation, tissues were first mechanically homogenized on ice and digested for 20 min at 37 °C with a digestion medium containing 250 U/ml collagenase (Sigma Aldrich, C5138, Stockholm, Sweden) and 50 U/ml DNase I (Sigma Aldrich, D4527, Stockholm, Sweden) before filtering through a 70 μm cell strainer (BD Falcon, Stockholm, Sweden). Lung cells were next resuspended in 40% Percoll (GE Healthcare, Sweden), the pelleted cells were collected, erythrocytes were lysed (Red Blood Cell Lysis Buffer, Sigma, Stockholm, Sweden) and the obtained cells were extensively washed with PBS (HyClone, Nordic Biolabs, Täby, Sweden) containing 2% foetal bovine serum, FBS (HyClone, Nordic Biolabs, Täby, Sweden). Immunomagnetically enriched and FACS sorted lung and splenic ILC2s were either directly stained with fluorochrome-conjugated antibodies against CD45, KLRG1 and ST2 for FACS analysis or seeded in cell culture plates (0.8 × 10^4^ cells/ml) and cultured in the presence of recombinant mouse (rm) IL-7 and rmIL-33 (10 ng/ml each) for 9 days at 37 °C, 5% CO_2_ (the cytokine-supplemented medium was changed every other day). Upon completion of that time period the cultured cells were stained for the same markers. Moreover, part of the IL25-induced splenic ILC2s (10^4^ cells/ml) were incubated with several recombinant mouse cytokine combinations supplemented in the cell culture medium (IL-25; IL-7 + IL-25; IL-7+ IL-25+ IL-33; IL-7 + IL-33 10 ng/ml each, R&D systems, Abingdon, UK) for the same time period and then stained for CD45, KLRG1 and ST2.

The number of mice included in each experiment is shown in the figures as single dots. All assessments of outcomes were performed blinded.

### Flow cytometry

Single cell suspensions of splenocytes were obtained by pressing the organs through a 70-μm cell strainer (BD Falcon, Stockholm, Sweden). Peritoneal cells were obtained through centrifugation of the obtained peritoneal fluid. Erythrocytes were lysed using red blood cell lysing buffer (Sigma, Stockholm, Sweden). Upon extensive washing, peritoneal cells were directly stained with fluorochrome-conjugated antibodies for flow cytometric analysis. The number of mice that were included in the FACS analysis concerning peritoneal cells varied due to the restriction of the number of cells that could be obtained by peritoneal lavage (Fig. 2). Part of the splenocytes were cultured in culture medium (RPMI 1640 medium containing 10% heat-inactivated FBS (HyClone, Nordic Biolabs, Täby, Sweden), 1 mmol/L sodium pyruvate, 10 mmol/L Hepes, 50 U penicillin, 50 μg/mL streptomycin, 0.05 mmol/L β-mercaptoethanol and 2 mmol/L L-glutamine; GIBCO, Life Technologies, Stockholm, Sweden) and used for cytokine secretion analysis while the rest of the cells were directly used for flow cytometric analysis. Peritoneal and spleen cells were stained with fluorochrome conjugated antibodies as previously described [25]; SiglecF-PE (clone E50–2440, BD Biosciences, Stockholm, Sweden) Arginase 1-APC (IC5868A), IL17-RB-APC (clone 752,101) and ST2-AF700 (clone 245,707) all from R&D systems (Abingdon, UK), Lin-biotinylated (Stem cell Technologies Inc., Grenoble, France, Lineage antibody cocktail: CD3, CD4, CD8, CD11b, CD11c, CD45R, CD19, Gr-1, FcεRI, NK 1.1 and Ter-119), IgM-FITC (eB121-15F9, eBioscience, Fisher Scientific, Göteborg, Sweden), CD11c-PE/Cy7 (clone N418), CD11b-PB (clone, M1/70), CD45-APC/Cy7 (clone 30-F11), F4/80-FITC (clone BM8), CD127-FITC (clone SB/199), ICOS-PB (clone C398.4A), B220-PE/Cy7 (clone RA3-6B2), CD43-APC (clone S11), CD19-AF700 (clone 6D5), forkhead box P3 (FoxP3)-PE (clone MF-14), CD3-PE/Cy7 (clone 145-2C11), CD4-PB (clone RM4–4), CD25-APC (clone 3C7), IL-5-APC (clone TRFK5), KLRG1-PE (clone 2F1/KLRG1), Streptavidin-PE/Cy7 all from Biolegend (London, UK). Cells intended for intracellular staining of IL-5 were stimulated with 20 ng/mL PMA, 1 μg/mL Ionomycin and 5 μg/mL Brefeldin A (all purchased from Sigma-Aldrich, Stockholm, Sweden) for 4 h prior to staining. All flow cytometric data were analyzed with FlowJo software (Tree Star, Inc).

### Immunohistochemistry

Dissected BCA and hearts were embedded in OCT (Optimal Cutting Temperature; Tissue-Tek) and serial sections of 10 μm thickness were collected and frozen at -20 °C. *En face* preparations of aortas were dipped in 78% methanol and stained for 40 min in 0.16% Oil Red O dissolved in 78% methanol containing 0.22 mol/L NaOH. Regarding BCA specimens, sections where the biggest plaques were detected were utilized for assessment of each set of immuno−/histochemical target-staining. The number of mice that were used in this series of analyses were *n* = 6 for control group and *n* = 6 for the ILC2 group. For the rest of the mice that were not included either no plaques were observed in BCA specimens or the quality of the obtained sections was not satisfactory for immuno−/ histochemistry analyses. For Oil Red O staining, heart and BCA sections were dipped in 0.5% v/w Oil Red O solution in 99% isopropylalcohol for 10 min, washed in distilled H_2_0 and stained in Mayer’s hematoxylin solution (Sigma-Aldrich, Stockholm, Sweden) for 2–3 min followed by a final washing step. CD68 (rabbit anti-mouse ab 125,212, Abcam, Cambridge, UK), IgM (Biotinylated anti-mouse IgM, BA 2020, Vector Lab, Bionordika, Stockholm, Sweden), arginase 1 (goat anti-mouse ab 60,176, Abcam, Cambridge, UK), CD3 (anti-CD3, Dako A0452, Glostrup, Denmark), Siglec F (rat anti mouse 14–1702, clone 1RNM44N, eBioscience, Fisher Scientific, Göteborg, Sweden), α actin smooth muscle cell (αSMactin, clone 1A4, Sigma A2547, Stockholm, Sweden) content were assessed with the use of the respective antibodies specified in parentheses while DAB detection kit was used for color development (Vector Lab, Bionordika, Stockholm, Sweden) and the sections were counterstained in haematoxylin. Negative controls used; CD68 (isotype ctrl, rabbit ab 172,730), arginase I (isotype ctrl, goat IgG ab 37,373), Siglec F (isotype ctrl, IgG2a, ab 18,450), α actin smooth muscle cell content (rabbit IgG, clone EPR25A, ab172730) all from Abcam (Cambridge, UK), CD3 (Dako X0936, Glostrup, Denmark). The collagen content of the plaques was assessed by Van Gieson Solution Acid Fuchsin staining (Sigma-Aldrich, Stockholm, Sweden). Necrotic core areas were assessed upon hematoxylin/eosin staining of sectioned tissues and evaluated as acellular areas > 3000 μm^2^. All stainings were obtained with Scan Scope CS system (LRI Instrument AB) and quantified with BioPix iQ 2.3.1 software.

### Cytokine analysis

Splenocytes and peritoneal cells (1 × 10^6^ cells/well) were cultured in the presence and absence of 1 μg/mL Ionomycin and 20 ng/mL PMA for 24 h. Cell culture supernatants were frozen at -80 °C until further analysis. Plasma cytokine levels as well as cytokine release by cells, were assessed using Luminex xMAP technology according to the company’s instructions (IL-1β, IL-2, IL-4, IL-5, IL-6, IL-9, IL-10, IL-12(p70), IL-13, IL-17A, Eotaxin, IFNγ, GM-CSF, Bio Rad, Solna, Sweden).

### Immunoglobulins

Plasma IgA, IgG1, IgG2a, IgG2b and IgM levels were assessed with the use of Mouse Isotyping Panel 1 Assay kit from Mesoscale according to the company’s instructions (MSD Multi-spot Assays, Mesoscale Discovery, Rockville, Maryland). Plasma IgM antibodies targeting PC were detected with an ELISA kit from Athera Biotechnologies (Athera Biotechnologies AB, Solna, Sweden) according to the company’s instructions, with the exception of using peroxidase-conjugated anti-mouse IgM (Jackson ImmunoResearch, Novakemi, Handen, Sweden).

### Lipid analysis

Lipids in plasma were assessed with the use of Thermo Scientific™ Triglycerides and Cholesterol Reagents (Fisher Scientific, Göteborg, Sweden) while the standard curve was assessed by employing the Chemistry Calibrator from Pointe Scientific INC (Canton, MI). All procedures were performed according to the companies’ instructions. LDL/VLDL and HDL cholesterol levels were assessed with the use of a cholesterol assay kit (abcam, Cambridge, UK).

### Statistics

Data are presented as mean ± standard deviation. Unpaired *t* test was used for normally and Mann Whitney *U* test for non-normally distributed variables. Analysis was performed using GraphPad Prism 7.03 (Graphpad Software) and a level of *P < 0.05 was considered significant. (**P < 0.01, ***P < 0.001, ****P < 0.0001).

## Supplementary information

**Additional file 1.** Gating strategy of FACS-sorted ILC2s and study design of ILC2 transfers to apoE^−/−^ mice. ApoE^−/−^ mice were injected subcutaneously with rmIL-25 (1 μg/day) for 7 days. Spleens were dissected, single cell suspensions of splenocytes were prepared and ILC2s were enriched by immunomagnetic exclusion of Lineage^+^ cells. The cells were next stained with fluorochrome conjugated antibodies for FACS-sorting. IL25-induced ILC2s were identified as Lin^−^CD45^+^IL17RB^+^ICOS^+^IL7ra^int^ cells (a). The sorted cells were expanded in vitro in the presence of IL-7 and IL-33 until transfer to apoE^−/−^ mice. (b) ApoE^−/−^ mice (7–8 weeks old) were placed on high fat diet (HFD) for 9 weeks. The mice received serial ILC2 transfers (0.5 × 10^6^ cells/transfer/mouse) or equal volume of PBS as control, 2-weeks apart from each other until euthanasia at 16–17 weeks of age.

**Additional file 2. ** Cytokine secretion levels of peritoneal cells from apoE^−/−^ mice that received ILC2s. Peritoneal cells obtained from apoE^−/−^ mice that received serial transfers of ILC2s or PBS as control were stimulated in vitro in the presence of PMA and Ionomycin for 24 h. Cytokine levels were assessed in the supernatants of the cultured cells. Data are presented as Mean ± Standard Deviation, Mann-Whitney *U* test. IL, interleukin; IFNγ, interferon gamma.

**Additional file 3. ** Cytokine secretion levels of splenocytes from apoE^−/−^ mice that received ILC2s. Single-cell suspensions of splenocytes extracted from apoE^−/−^ mice that received serial transfers of ILC2s or PBS as control were stimulated in vitro in the presence of PMA and Ionomycin for 24 h. Cytokine levels were assessed in the supernatants of the cultured cells. Data are presented as Mean ± Standard Deviation, Mann-Whitney *U* test. IL, interleukin; GM-CSF, granulocyte-macrophage colony-stimulating factor; IFNγ, interferon gamma.

**Additional file 4. ** Plasma cytokine levels of apoE^−/−^ mice that received ILC2s. Plasma cytokine levels of apoE^−/−^ mice that received serial ILC2 transfers or equal volume of PBS as control. Data are presented as Mean ± Standard Deviation, Mann-Whitney *U* test. IL, interleukin; GM-CSF, granulocyte-macrophage colony-stimulating factor; IFNγ, interferon gamma.

**Additional file 5. ** Plasma immunoglobulin levels of apoE^−/−^ mice that received ILC2s. Plasma immunoglobulin levels in the plasma of apoE^−/−^ mice that received serial transfers of ILC2s or PBS as control. Data are presented as Mean ± Standard Deviation, Mann-Whitney *U* test. Ig, immunoglobulin.

**Additional file 6.** Assessment of necrotic cores in subvalvular heart sections of apoE^−/−^ mice that received ILC2s. Quantification of necrotic core areas (a) and respective percentages (b) of total plaque areas in hematoxylin/eosin stained subvalvular heart sections of apoE^−/−^ mice fed a high fat diet for 9 weeks. The mice received 4 i.p. ILC2 transfers (0.5 × 10^6^ cells/transfer) or equal volume of PBS during that time period until euthanasia at 16–17 weeks of age. Necrotic core areas were assessed as acellular regions of > 3000 μm^2^. Each data point represents one mouse.

**Additional file 7.** Plasma lipid levels of apoE^−/−^ mice that received ILC2s. Plasma (a) total cholesterol, (b) LDL/VLDL cholesterol, (c) HDL cholesterol (d) triglyceride levels and (e) weight of apoE^−/−^ mice upon euthanasia at 16–17 weeks of age. The mice were fed a high fat diet for 9 weeks and received 4 i.p. ILC2 transfers (0.5 × 10^6^ cells/transfer) or equal volume of PBS during that time period. Each data point represents one mouse.

**Additional file 8.** Plaque composition of subvalvular heart sections of apoE^−/−^ mice that received ILC2s. Immunohistochemical analyses of subvalvular heart sections from apoE^−/−^ mice, fed a high fat diet that received 4 i.p. ILC2 transfers (0.5 × 10^6^ cells/transfer) or equal volume of PBS. Quantifications of a) CD68^+^ macrophage, b) collagen, c) αSMactin^+^ smooth muscle cell, d) CD3^+^ T cell, e) Arginase 1^+^, f) IgM^+^ content are depicted as a percentage of total plaque area. Each data point represents one mouse.

**Additional file 9.** Plaque composition of brachiocephalic artery (BCA) sections of apoE^−/−^ mice that received ILC2s. Immunohistochemical analyses of BCA sections from apoE^−/−^ mice, fed a high fat diet that received 4 i.p. ILC2 transfers (0.5 × 10^6^ cells/transfer) or equal volume of PBS. Quantifications of a) CD68^+^ macrophage, b) CD3^+^ T cell, c) αSMactin^+^ smooth muscle cell, d) IgM^+^ content are depicted as a percentage of total plaque area. Each data point represents one mouse.

## Data Availability

The datasets used and/or analysed during the current study are available from the corresponding author on reasonable request.

## Abbreviations

AAMs: Alternatively activated macrophages
apoB: Apolipoprotein B
apoE^−/−^: Apolipoprotein E deficient
Arg1: Arginase 1
BCA: Brachiocephalic artery
CD: Cluster of differentiation
FACS: Fluorescence activated cell sorter
FoxP3: Forkhead box P3
GM-CSF: Granulocyte macrophage colony-stimulating factor
HFD: High fat diet
i.p.: Intraperitoneal
ICOS: Inducible costimulatory molecule
IFNγ: Interferon γ
Ig: Immunoglobulin
iILC2s: Inflammatory ILC2s
IL: Interleukin
IL17RB: Interleukin 17 receptor B
IL-25: Interleukin-25
IL7ra: Interleukin 7 receptor a
ILC2s: type 2 innate lymphoid cells
KLRG1: Killer-cell lectin like receptor G1
LDL: Low-density-lipoprotein
Lin: Lineage
nILC2s: Natural ILC2s
ORO: Oil Red O
oxLDL: oxidized-LDL
PBS: Phosphate-buffered saline
PC: anti-phosphorylcholine
PMA: Phorbol 12-myristate 13-acetate
ST2: Suppression of tumorigenicity 2
Th1: T helper 1
Treg: T regulatory
Tregs: T regulatory cells
VAT: Visceral adipose tissue
w: Week
αSMactin: α actin smooth muscle cell
