# Supplementary figures and images for: ILC2 transfers to apolipoprotein E deficient mice reduce the lipid content of atherosclerotic lesions
Source: BMC Immunol. 2019 Dec 10;20:47. doi: 10.1186/s12865-019-0330-z (PMC6905041; doi:10.1186/s12865-019-0330-z)

Additional file 1


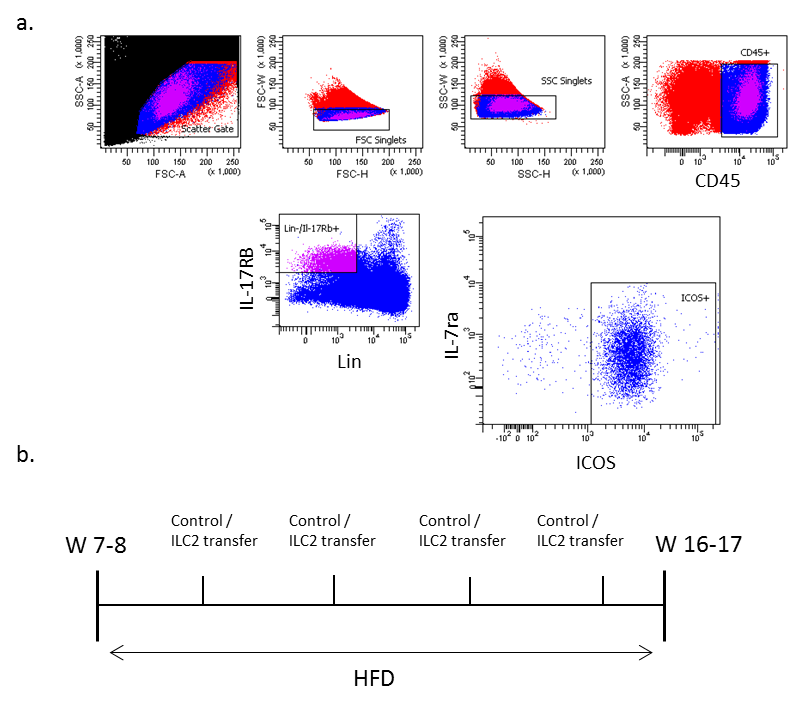

Supplement: Supplementary file 1 — Additional file 1. Gating strategy of FACS-sorted ILC2s and study design of ILC2 transfers to apoE−/− mice. ApoE−/− mice were injected subcutaneously with rmIL-25 (1 μg/day) for 7 days. Spleens were dissected, single cell suspensions of splenocytes were prepared and ILC2s were enriched by immunomagnetic exclusion of Lineage+ cells. The cells were next stained with fluorochrome conjugated antibodies for FACS-sorting. IL25-induced ILC2s were identified as Lin−CD45+IL17RB+ICOS+IL7raint cells (a). The sorted cells were expanded in vitro in the presence of IL-7 and IL-33 until transfer to apoE−/− mice. (b) ApoE−/− mice (7–8 weeks old) were placed on high fat diet (HFD) for 9 weeks. The mice received serial ILC2 transfers (0.5 × 10^6^ cells/transfer/mouse) or equal volume of PBS as control, 2-weeks apart from each other until euthanasia at 16–17 weeks of age. [file 12865_2019_330_MOESM1_ESM.doc]

Additional file 6


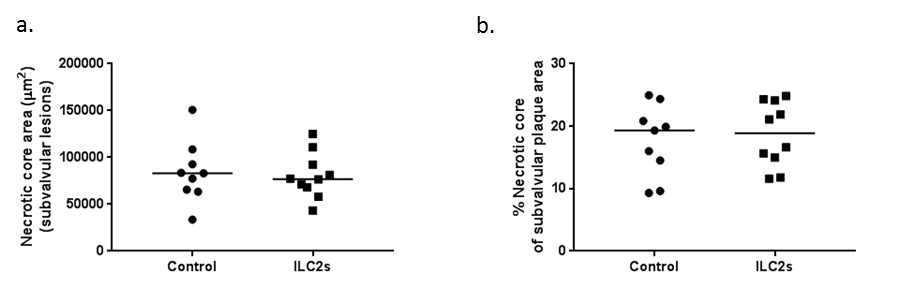

Supplement: Supplementary file 6 — Additional file 6. Assessment of necrotic cores in subvalvular heart sections of apoE−/− mice that received ILC2s. Quantification of necrotic core areas (a) and respective percentages (b) of total plaque areas in hematoxylin/eosin stained subvalvular heart sections of apoE−/− mice fed a high fat diet for 9 weeks. The mice received 4 i.p. ILC2 transfers (0.5 × 10^6^ cells/transfer) or equal volume of PBS during that time period until euthanasia at 16–17 weeks of age. Necrotic core areas were assessed as acellular regions of > 3000 μm2. Each data point represents one mouse. [file 12865_2019_330_MOESM6_ESM.doc]

Additional file 7


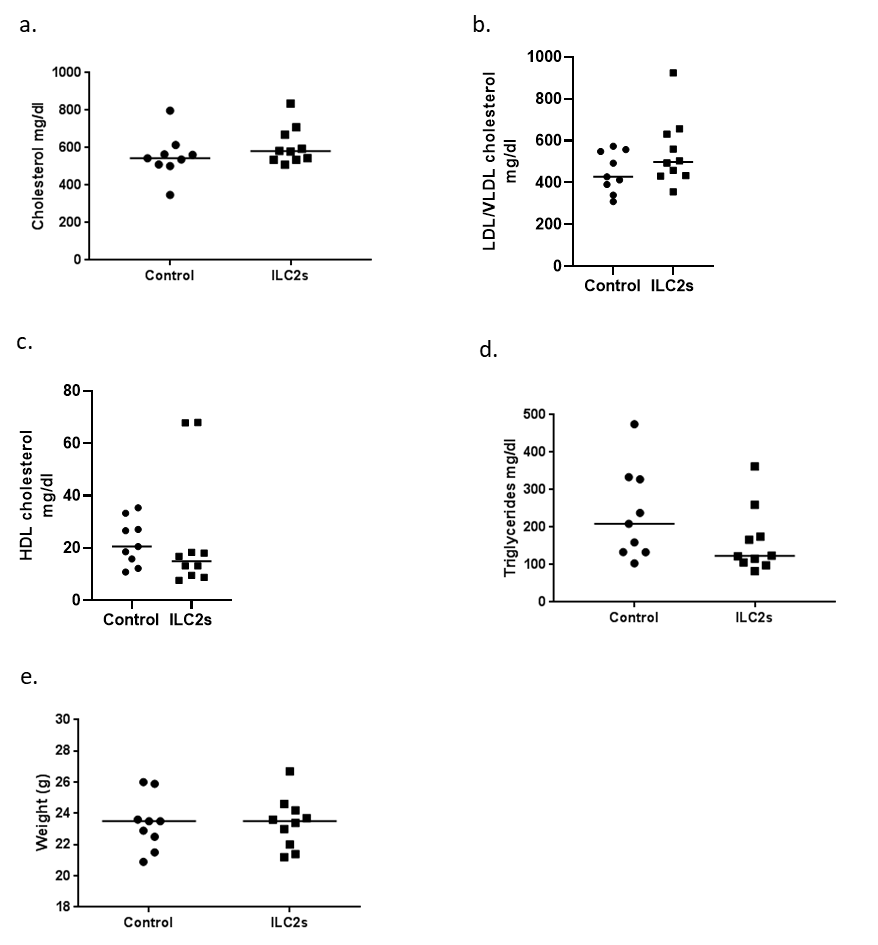

Supplement: Supplementary file 7 — Additional file 7. Plasma lipid levels of apoE−/− mice that received ILC2s. Plasma (a) total cholesterol, (b) LDL/VLDL cholesterol, (c) HDL cholesterol (d) triglyceride levels and (e) weight of apoE−/− mice upon euthanasia at 16–17 weeks of age. The mice were fed a high fat diet for 9 weeks and received 4 i.p. ILC2 transfers (0.5 × 10^6^ cells/transfer) or equal volume of PBS during that time period. Each data point represents one mouse. [file 12865_2019_330_MOESM7_ESM.doc]

Additional file 8


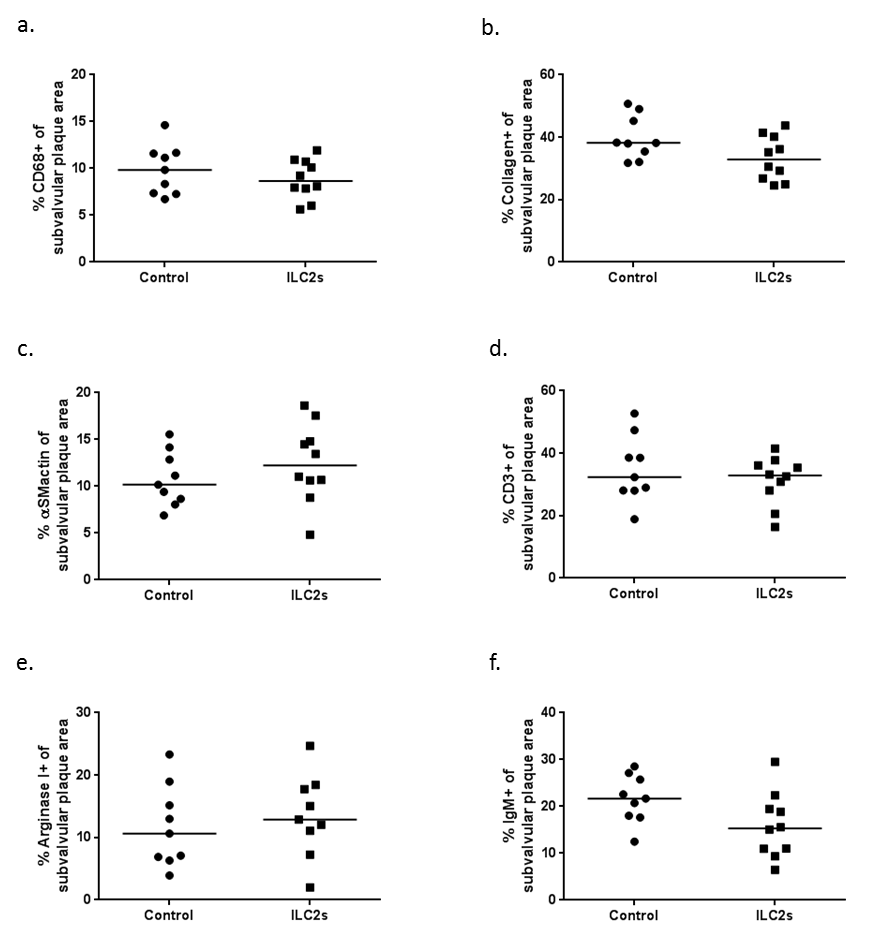

Supplement: Supplementary file 8 — Additional file 8. Plaque composition of subvalvular heart sections of apoE−/− mice that received ILC2s. Immunohistochemical analyses of subvalvular heart sections from apoE−/− mice, fed a high fat diet that received 4 i.p. ILC2 transfers (0.5 × 10^6^ cells/transfer) or equal volume of PBS. Quantifications of a) CD68+ macrophage, b) collagen, c) αSMactin+ smooth muscle cell, d) CD3+ T cell, e) Arginase 1+, f) IgM+ content are depicted as a percentage of total plaque area. Each data point represents one mouse. [file 12865_2019_330_MOESM8_ESM.doc]

Additional file 9


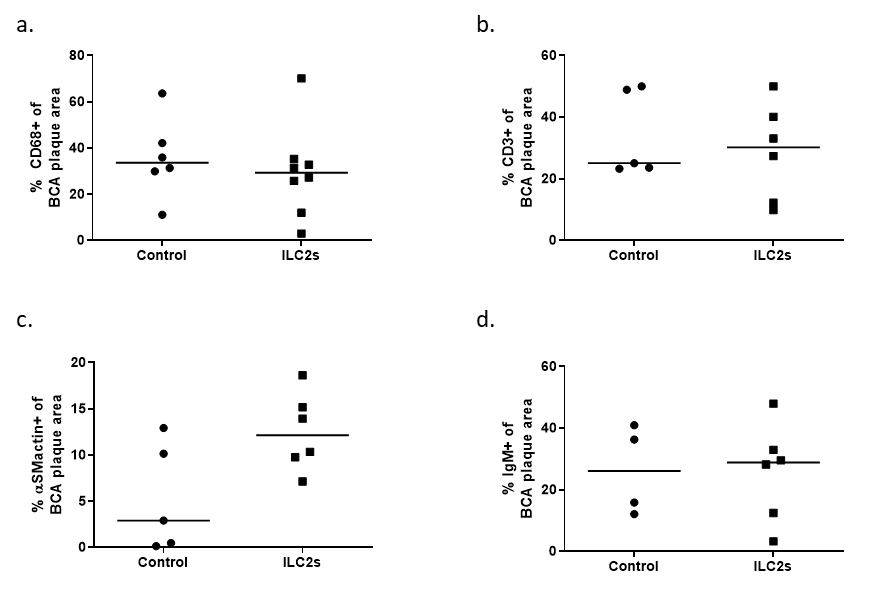

Supplement: Supplementary file 9 — Additional file 9. Plaque composition of brachiocephalic artery (BCA) sections of apoE−/− mice that received ILC2s. Immunohistochemical analyses of BCA sections from apoE−/− mice, fed a high fat diet that received 4 i.p. ILC2 transfers (0.5 × 10^6^ cells/transfer) or equal volume of PBS. Quantifications of a) CD68+ macrophage, b) CD3+ T cell, c) αSMactin+ smooth muscle cell, d) IgM+ content are depicted as a percentage of total plaque area. Each data point represents one mouse. [file 12865_2019_330_MOESM9_ESM.doc]
