# Supplementary material for: ILC2 transfers to apolipoprotein E deficient mice reduce the lipid content of atherosclerotic lesions
Source: BMC Immunol. 2019 Dec 10;20:47. doi: 10.1186/s12865-019-0330-z (PMC6905041; doi:10.1186/s12865-019-0330-z)
Supplement: Supplementary file 2 — Additional file 2. Cytokine secretion levels of peritoneal cells from apoE−/− mice that received ILC2s. Peritoneal cells obtained from apoE−/− mice that received serial transfers of ILC2s or PBS as control were stimulated in vitro in the presence of PMA and Ionomycin for 24 h. Cytokine levels were assessed in the supernatants of the cultured cells. Data are presented as Mean ± Standard Deviation, Mann-Whitney U test. IL, interleukin; IFNγ, interferon gamma. [file 12865_2019_330_MOESM2_ESM.doc]

Additional file 2

| Cytokine (pg/ml) | Control (*n*=7) | ILC2s (*n*=10) | *P* |
| --- | --- | --- | --- |
| IL-1β | 87.6 ± 14.6 | 84.3 ± 10.1 | 0.62 |
| IL-5 | 629.0 ± 478.4 | 651.6 ± 433.5 | 0.89 |
| IL-6 | 3556 ± 2633 | 6759 ± 5056 | 0.36 |
| IL-9 | 88.9 ± 9.2 | 78.9 ± 11.8 | 0.12 |
| IL-10 | 158.3 ± 84.1 | 138.9 ± 42.0 | 0.88 |
| IL-12(p70) | 81.7 ± 20.9 | 72.4 ± 17.5 | 0.79 |
| IL-13 | 285.9 ± 80.5 | 403.9 ± 199.0 | 0.37 |
| Eotaxin | 752.7 ± 196.8 | 695.3 ± 127.1 | 0.43 |
| IFNγ | 1511.0 ± 541.8 | 1139.0 ± 537.0 | 0.31 |
