# Supplementary material for: ILC2 transfers to apolipoprotein E deficient mice reduce the lipid content of atherosclerotic lesions
Source: BMC Immunol. 2019 Dec 10;20:47. doi: 10.1186/s12865-019-0330-z (PMC6905041; doi:10.1186/s12865-019-0330-z)
Supplement: Supplementary file 3 — Additional file 3. Cytokine secretion levels of splenocytes from apoE−/− mice that received ILC2s. Single-cell suspensions of splenocytes extracted from apoE−/− mice that received serial transfers of ILC2s or PBS as control were stimulated in vitro in the presence of PMA and Ionomycin for 24 h. Cytokine levels were assessed in the supernatants of the cultured cells. Data are presented as Mean ± Standard Deviation, Mann-Whitney U test. IL, interleukin; GM-CSF, granulocyte-macrophage colony-stimulating factor; IFNγ, interferon gamma. [file 12865_2019_330_MOESM3_ESM.doc]

Additional file 3

| Cytokine (pg/ml) | Control (*n*=9) | ILC2s (*n*=10) | *P* |
| --- | --- | --- | --- |
| IL-1β | 83.9 ± 6.0 | 81.4 ± 11.8 | 0.36 |
| IL-2 | 53601 ± 32768 | 218198 ± 255867 | 0.32 |
| IL-4 | 206.7 ± 49.0 | 172.3 ± 59.3 | 0.07 |
| IL-6 | 159.0 ± 97.8 | 147.3 ± 36.1 | 0.60 |
| IL-9 | 132.8 ± 18.6 | 144.1 ± 30.0 | 0.73 |
| IL-10 | 135.5 ± 51.0 | 100.2 ± 56.6 | 0.08 |
| IL-12(p70) | 35.1 ± 7.6 | 37.3 ± 7.5 | 0.54 |
| IL-13 | 339.5 ± 155.4 | 382.0 ± 117.5 | 0.27 |
| IL-17 | 671.4 ± 533.3 | 594.9 ± 315.7 | 0.91 |
| Eotaxin | 717.6 ± 122.1 | 657.9 ± 151.8 | 0.46 |
| GM-CSF | 374.3 ± 120.0 | 452.1 ± 152.9 | 0.13 |
| IFNγ | 6446 ± 2024 | 9913 ± 7133 | 0.22 |
