# Supplementary material for: ILC2 transfers to apolipoprotein E deficient mice reduce the lipid content of atherosclerotic lesions
Source: BMC Immunol. 2019 Dec 10;20:47. doi: 10.1186/s12865-019-0330-z (PMC6905041; doi:10.1186/s12865-019-0330-z)
Supplement: Supplementary file 4 — Additional file 4. Plasma cytokine levels of apoE−/− mice that received ILC2s. Plasma cytokine levels of apoE−/− mice that received serial ILC2 transfers or equal volume of PBS as control. Data are presented as Mean ± Standard Deviation, Mann-Whitney U test. IL, interleukin; GM-CSF, granulocyte-macrophage colony-stimulating factor; IFNγ, interferon gamma. [file 12865_2019_330_MOESM4_ESM.doc]

Additional file 4

| Cytokine (pg/ml) | Control (*n*=9) | ILC2s (*n*=10) | *P* |
| --- | --- | --- | --- |
| IL-1β | 1090 ± 356 | 1047 ± 243 | 0.72 |
| IL-2 | 102.2 ± 65.0 | 83.3 ± 46.3 | 0.56 |
| IL-4 | 80.7 ± 63.6 | 64.2 ± 18.3 | 0.55 |
| IL-5 | 184.2 ± 104.9 | 168.5 ± 50.8 | 0.95 |
| IL-6 | 77.2 ± 29.1 | 76.5 ± 39.0 | 0.66 |
| IL-9 | 626.7 ± 334.4 | 808.3 ± 199.9 | 0.10 |
| IL-10 | 351.6 ± 108.8 | 350.9 ± 105.1 | 0.83 |
| IL-12(p70) | 774.5 ± 274.1 | 759.2 ± 251.3 | 0.99 |
| IL-13 | 1785.0 ± 400.1 | 1815.0 ± 329.1 | >0.99 |
| IL-17 | 272.6 ± 86.1 | 284.0 ± 121.1 | 0.89 |
| Eotaxin | 6834 ± 2131 | 7435 ± 2235 | 0.71 |
| GM-CSF | 291.2 ± 106.8 | 260.7 ± 102.6 | 0.56 |
| IFNγ | 142.5 ± 47.7 | 135.5 ± 29.7 | 0.72 |
