# Supplementary material for: ILC2 transfers to apolipoprotein E deficient mice reduce the lipid content of atherosclerotic lesions
Source: BMC Immunol. 2019 Dec 10;20:47. doi: 10.1186/s12865-019-0330-z (PMC6905041; doi:10.1186/s12865-019-0330-z)
Supplement: Supplementary file 5 — Additional file 5. Plasma immunoglobulin levels of apoE−/− mice that received ILC2s. Plasma immunoglobulin levels in the plasma of apoE−/− mice that received serial transfers of ILC2s or PBS as control. Data are presented as Mean ± Standard Deviation, Mann-Whitney U test. Ig, immunoglobulin. [file 12865_2019_330_MOESM5_ESM.doc]

Additional file 5

| Immunoglobulin (μg/ml) | Control (*n*=9) | ILC2s (*n*=10) | *P* |
| --- | --- | --- | --- |
| IgA | 70.8 ± 36.5 | 75.1 ± 37.6 | 0.91 |
| IgG1 | 328.7 ± 134.1 | 448.4 ± 300.1 | 0.60 |
| IgG2a | 25.1 ± 14.6 | 23.8 ± 8.2 | 0.89 |
| IgG2b | 962.6 ± 574.5 | 868.1 ± 340.3 | 0.72 |
| IgM | 809.9 ± 452.9 | 905.8 ± 445.9 | 0.72 |
